# Enhancement of FAK alleviates ventilator-induced alveolar epithelial cell injury

**DOI:** 10.1038/s41598-019-57350-6

**Published:** 2020-01-15

**Authors:** Mingxing Fang, Na Liu, Xiaoguang Yao, Tieling Xu, Zhiyong Wang

**Affiliations:** 1 Department of Intensive Care Medicine, The Third Hospital of HeBei Medical University, Shijiazhuang, China; 2 Department of Emergency, The fourth Hospital of HeBei Medical University, Shijiazhuang, China; 3 College of Integrative Medicine, Hebei University of Chinese Medicine, Shijiazhuang, China; 4 Department of Emergency, Hebei General Hospital, Shijiazhuang, China

**Keywords:** Respiratory distress syndrome, Molecular medicine

## Abstract

Mechanical ventilation induces lung injury by damaging alveolar epithelial cells (AECs), but the pathogenesis remains unknown. Focal adhesion kinase (FAK) is a cytoplasmic protein tyrosine kinase that is involved in cell growth and intracellular signal transduction pathways. This study explored the potential role of FAK in AECs during lung injury induced by mechanical ventilation. High-volume mechanical ventilation (HMV) was used to create a mouse lung injury model, which was validated by analysis of lung weight, bronchoalveolar lavage fluid and histological investigation. The expression of FAK and Akt in AECs were evaluated. In addition, recombinant FAK was administered to mice via the tail vein, and then the extent of lung injury was assessed. Mouse AECs were cultured *in vitro*, and FAK expression in cells under stretch was investigated. The effects of FAK on cell proliferation, migration and apoptosis were investigated. The results showed that HMV decreased FAK expression in AECs of mice, while FAK supplementation attenuated lung injury, reduced protein levels/cell counts in the bronchoalveolar lavage fluid and decreased histological lung injury and oedema. The protective effect of FAK promoted AEC proliferation and migration and prevented cells from undergoing apoptosis, which restored the integrity of the alveoli through Akt pathway. Therefore, the decrease in FAK expression by HMV is essential for injury to epithelial cells and the disruption of alveolar integrity. FAK supplementation can reduce AEC injury associated with HMV.

## Introduction

Ventilator-induced lung injury (VILI) is known as acute lung injury induced by mechanical ventilation and is the most common complication of the treatment of acute respiratory distress syndrome. Mechanical ventilation with high tidal volumes increases lung inflation and then produces lung overdistension or barotrauma because the repetitive opening and closing of alveoli at a high pressure creates shear stress and then leads to the impairment of cell adjunction. Moreover, over-pressure stress at the pathological level leads to cell apoptosis, inflammatory response, barrier dysfunction and the decreased synthesis of extracellular matrix proteins through the regulation of gene expression^1^.

FAK is a cytoplasmic tyrosine kinase, and its gene is highly conserved, with over 90% sequence identity between human (chromosome 8) and mouse (chromosome 15)^2^. Activated FAK forms a complex with Src family kinases and then phosphorylates other proteins to regulate various cell events, such as apoptosis, migration, the immune response, cell differentiation, and cell shape. Multiple downstream signalling pathways of FAK have been identified, including PI3K-Akt-mTOR, ERK1/2, and JNK^3^. Previous studies have revealed that FAK plays an important role in the regulation of AECs. Unfried K *et al*. suggested that carbon nanoparticles can contribute to the proliferation of lung epithelial cells through the FAK-PI3K-Akt pathway^4^. Ding Q *et al*. suggested that FAK can inhibit apoptosis and promote the epithelial-myofibroblast plasticity of AECs^5^.

Therefore, we hypothesize that FAK and its related pathways play important roles in the damage to AECs induced by VILI. In this study, we created a VILI mouse model and then investigated FAK expression in AECs. We found that VILI can decrease FAK expression significantly *in vitro* and vivo, whereas supplementation with FAK attenuates injury induced by high-volume mechanical ventilation (HMV) via the Akt pathway. Furthermore, we found that FAK can promote AEC proliferation and adhesion and prevent cells from undergoing apoptosis, which promotes the regeneration of AECs after injury.

## Results

### A mouse model of mechanical ventilation-induced lung injury

**Figure 1:**
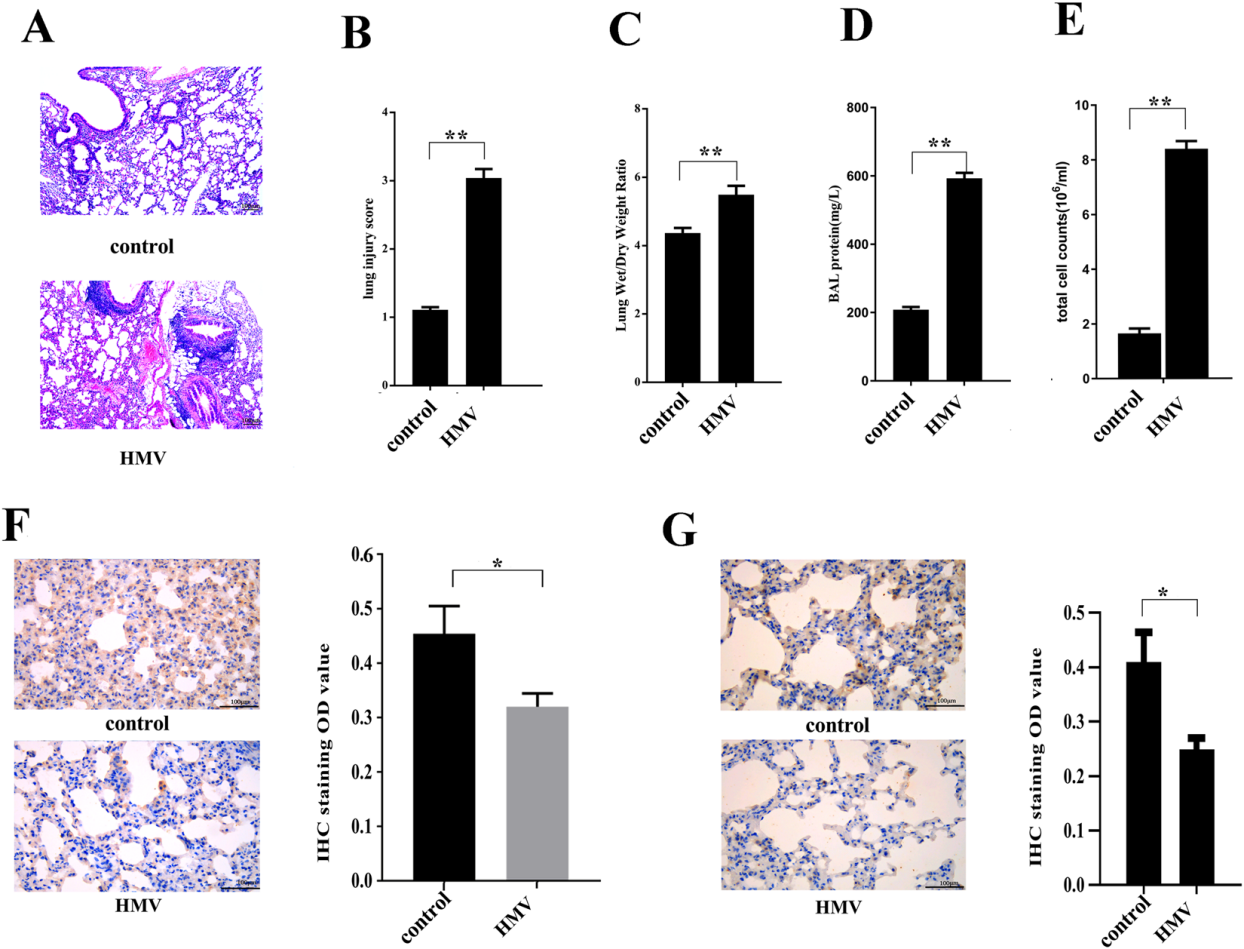
HMV induces lung injury in mice and reduces FAK expression in AECs. Treatment group mice underwent 4 h HMV (VT 30 ml/kg, respiratory rate of 75 breaths/min, positive end-expiratory pressure of 0 cm H2O), while control mice were maintained with spontaneous breathing. Then, lung permeability was assessed as representative images (**A**) of lung tissue (H&E, 100×) shown. The quantification of lung injury (**B**), the lung wet/dry ratio (**C**), BALF protein level (**D**) and BALF cell counts (**E**) in the harvested lung tissues. (**F**) HMV exposure reduced FAK expression in the mouse lung (IHC, 400×). (**G**) HMV exposure reduced phospho-Akt expression in the mouse lung (IHC, 400×). The data are presented as the mean ± SEM (*p < 0.05; **p < 0.01 by two-tailed t test). control, without mechanical ventilation; HMV, high-volume mechanical ventilation.

The mice underwent HMV for 4 h (tidal volume of 30 ml/kg, respiratory rate of 75 breaths/min, positive end-expiratory pressure of 0 cm H2O) and were then euthanized for the collection of lung tissues. Our data indicated that lung tissues from mice that underwent HMV treatment had a higher W/D ratio, more total cells and higher total protein content in the BALF than those of lung tissues from control mice that did not undergo mechanical ventilation. In lungs from mice that underwent HMV treatment, various pathological changes, including thickened alveolar walls, neutrophil infiltration, haemorrhage, and hyline membrane formation, were observed (Fig. 1), and the lung injury score in the HMV group was significantly higher than that in the control group. Therefore, the results indicated that HMV can induce lung injury and lung oedema *in vivo*. Furthermore, we evaluated the FAK expression level in AECs by IHC, and the results showed that HMV treatment, compared to control, clearly decreased FAK expression levels, which is consistent with some other studies^6,7^. Moreover, using flow cytometry, we collected primary AECs from mouse lung tissues at different timepoints (0, 8, 24 h) after HMV treatment, and then we investigated FAK expression in AECs by western blotting. The results indicated that FAK expression was decreased significantly compared to that in the untreated controls, whereas there was no significant difference in FAK expression among the different time points (Supplemental Fig. 1). In addition, the phosphorylation of Akt was decreased in AECs after HMV treatment.

### FAK prevents cells from undergoing apoptosis

**Figure 2:**
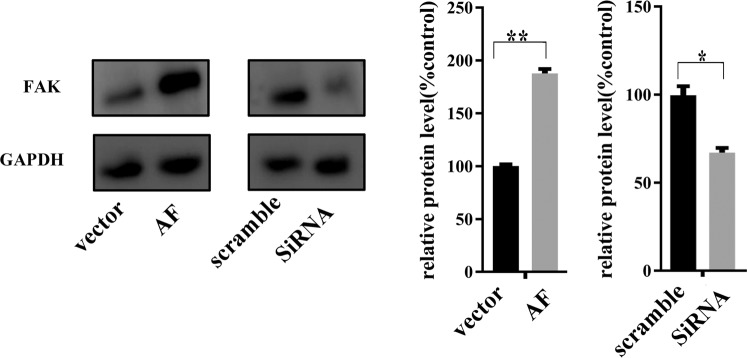
Transfection of FAK recombinant adenovirus (AF) increased FAK expression, whereas knocking down FAK by siRNA inhibited FAK expression in MLE-15 cells, as determined by western blotting (representative images and relative quantifications are shown). All experiments were performed in triplicate, and the data are presented as the mean ± SEM (*p < 0.05; **p < 0.01 by two-tailed t test).

Thereafter, MLE-15 cells stably expressing FAK and FAK knockdown cells were generated for further experiments and validated by western blotting (Fig. 2).

**Figure 3:**
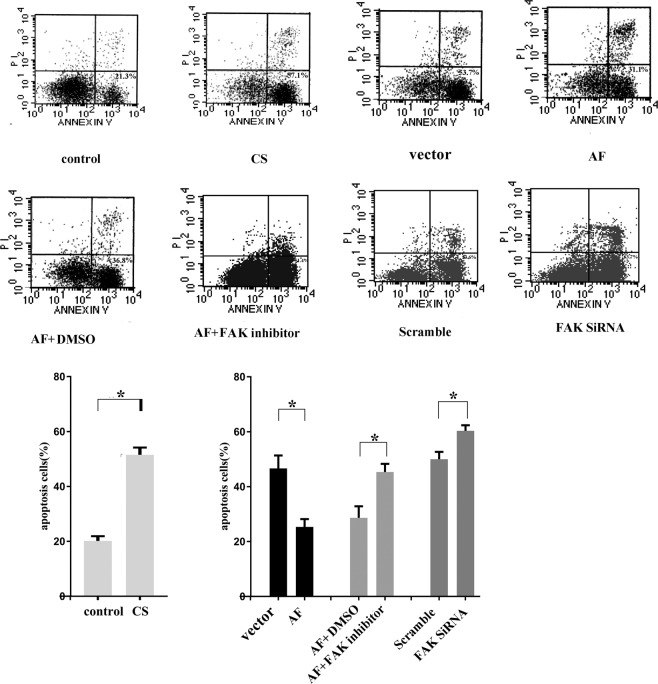
FAK prevents apoptosis of MLE-15 cells *in vitro* under CS conditions. Cells were transfected with ectopic FAK (FAK recombinant adenovirus (AF)) or FAK siRNA, treated with a FAK inhibitor or left untreated. Thereafter, the cells were exposed to CS conditions for 4 h, collected, stained with annexin V and PI and analysed by FACS. The number of apoptotic cells (Annexin V-positive cells) was indicated as the percentage of gated cells. Representative images and relative quantifications are shown. The results indicate that CS treatment promoted the apoptosis of MLE-15 cells. And the pro-apoptotic effect of CS was attenuated by FAK expression, while FAK knockdown promoted cell apoptosis. Moreover, the anti-apoptotic effect of FAK was blocked by a FAK inhibitor. All experiments were performed in triplicate, and the data are presented as the mean ± SEM (*p < 0.05; **p < 0.01 by two-tailed t test). control: no cell stretch treatment; CS: cell stretch treatment only; vector: transfection with vector followed by cell stretch treatment; AF: transfection with FAK recombinant adenovirus followed by cell stretch treatment; AF + DMSO: transfection with FAK recombinant adenovirus and treatment with DMSO followed by cell stretch treatment; AF + FAK inhibitor: transfection with FAK recombinant adenovirus and treatment with a FAK inhibitor followed by cell stretch treatment; scramble: transfection with scramble probe followed by cell stretch treatment; FAK siRNA: transfection with FAK siRNA followed by cell stretch treatment.

To recapitulate the outcomes of VILI in cells *in vitro*, we plated and maintained MLE-15 cells under CS conditions for 4 h (18% CS, 30 cycles/min). Thereafter, the cells were collected, stained with annexin V and PI and analysed by flow cytometry to evaluate apoptosis. We found that the number of apoptotic cells after CS treatment was more than 2-fold higher than that observed without CS treatment (Fig. 3A). In addition, MLE-15 cells received different pre-treatments, including ectopic FAK expression, siRNA transfection, and FAK inhibitor treatment, and were then cultured in CS conditions for 4 h. We found that FAK expression significantly reduced the percentage of apoptotic cells (20.33% compared to 52.16% in the controls without FAK ectopic expression, p = 0.001 (F statistic: F = 0.592, p = 0.485), Fig. 3A). The apoptosis rate was increased after CS treatment in cells in which FAK expression was blocked by siRNA compared to controls (62.45% vs 49.30%, p = 0.033 (F statistic: F = 0.308, p = 0.609)). Additionally, when the FAK-expressing cells were also treated with FAK inhibitor, the apoptotic cell percentage was significantly higher than that in cells not treated with the FAK inhibitor.

### FAK promotes cell migration and proliferation

**Figure 4:**
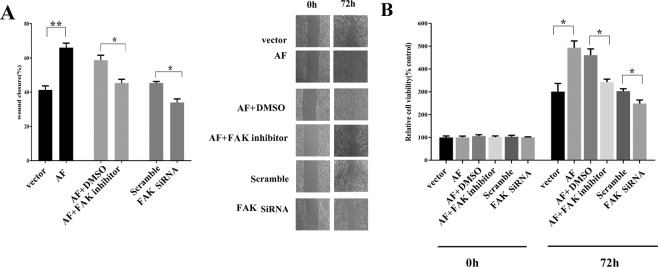
FAK promotes MLE-15 cell migration and proliferation *in vitro*. (**A**) A wound healing assay was performed as described in the methods, and images were acquired with a light microscope (representative images as shown). The graphs present the percentage wound recovery in cells that received different treatments after 72 h of cell migration. Ectopic FAK expression promoted wound closure, and this was blocked by a FAK inhibitor or FAK siRNA. (**B**) Cells received various pre-treatments and were then cultured for 72 h. Cell proliferation was measured using WST-1. The results showed that FAK overexpression increased cell proliferation compared to that in the controls transfected vector transfection, whereas a FAK inhibitor inhibited cell proliferation induced by AF. Additionally, FAK siRNA decreased cell proliferation. All results are representative of data from experiments performed in triplicate. The data are presented as the mean ± SEM (*p < 0.05; **p < 0.01 by two-tailed t test). The pre-treatments are as follows: control: no treatment; CS: cell stretch only; AF: ectopic FAK expression (FAK recombinant adenovirus transfection); siRNA: FAK knockdown; DMSO: treated with DMSO; FAK inhibitor: treated with a FAK inhibitor; scramble: treated with scramble probe.

To determine whether FAK plays a role in the migration and proliferation properties of cells, we measured the status of FAK-expressing and FAK knockdown MLE-15 cells. We found that ectopic expression of FAK promoted cell migration under CS conditions and that this effect was abrogated by a FAK inhibitor or FAK knockdown (Fig. 4A). In addition, we tested the effect of FAK on cell proliferation *in vitro*. FAK-expressing MLE-15 cells displayed a higher proliferation rate compared to that of the controls. However, in FAK-expressing cells simultaneously treated with a FAK inhibitor and in FAK knockdown cells, proliferation was inhibited significantly (Fig. 4B). Therefore, we suggest that FAK can promote AEC migration and proliferation and prevent cells from undergoing apoptosis.

It is already known that phosphorylated FAK can activate its downstream pathway and then plays a role in cell events. We investigated the level of phosphorylation of FAK in AECs under CS conditions using a phospho- FAK antibody (phosphorylated at Tyr397), and we detected a significant decrease in phosphorylated FAK in cells under CS conditions compared with control cells (Supplemental Fig. 2).

### The effect of FAK in AECs *in vivo*

**Figure 5:**
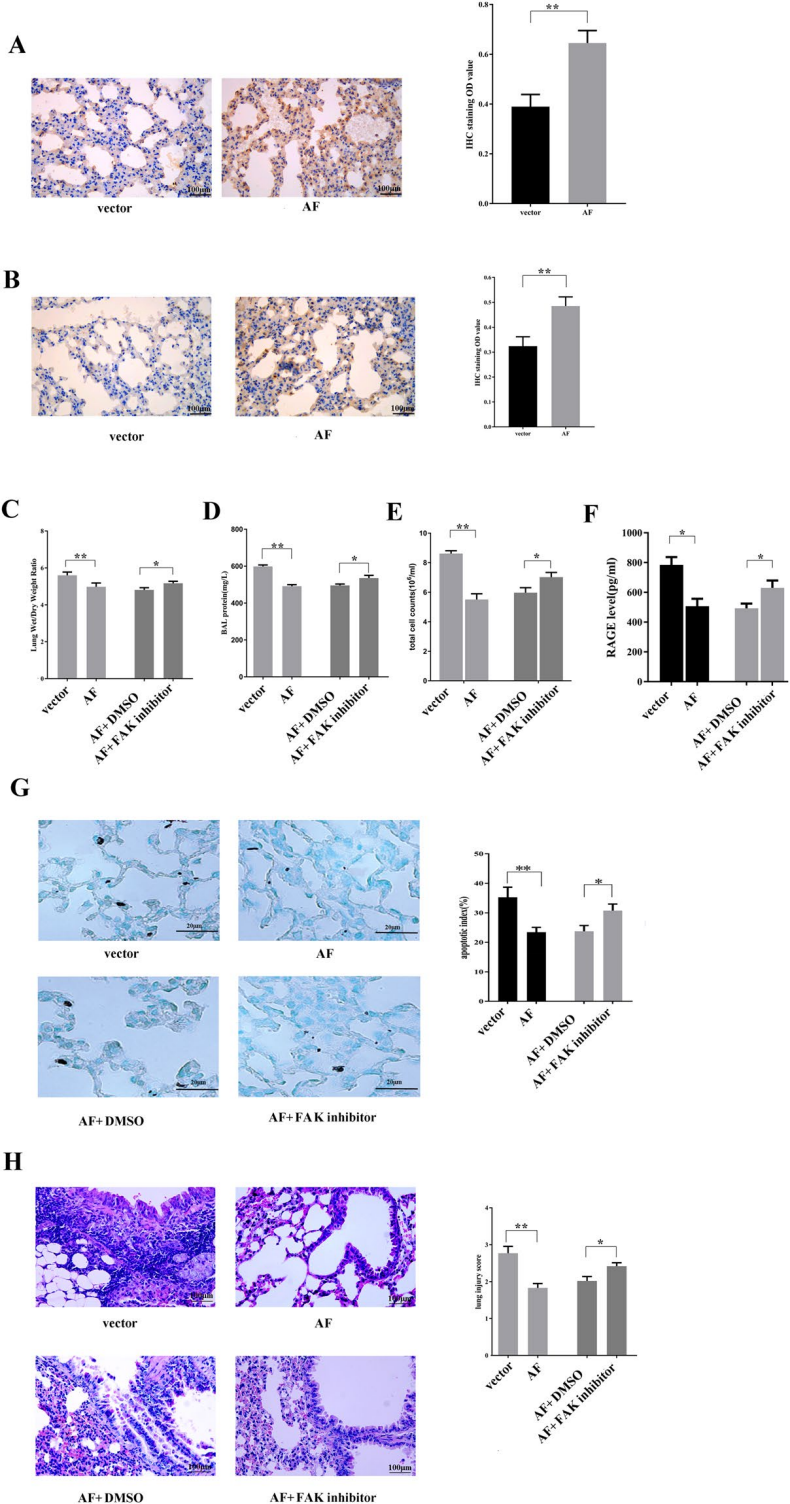
FAK supplementation decreases lung injury in mice challenged with 4 h of HMV. Representative images (IHC staining, 400×) and the relative semiquantitative analysis showing that FAK recombinant adenovirus (AF) injection enhanced the expression of FAK (**A**) and phospho-Akt (**B**) in AECs. The lung wet/dry ratio (**C**), BALF protein level (**D**), BALF cell counts (**E**) and RAGE level in the BALF (**F**) in the different groups of mice are shown. (**G**) Representative images (400×) of apoptosis in AECs, as visualised by TUNEL staining, and the quantification of the results. (**H**) Representative images (H&E, 400×) of lung tissue and the quantification of lung injury. FAK supplementation attenuated injury, inducing a decreased lung injury score and apoptosis index in lung tissues, as well as a lower protein level, RAGE level and cell counts in the BALF, whereas the protective effect of FAK was blocked by a FAK inhibitor. Lung injury was triggered by HMV, and mice received different treatments before HMV as follows: injection with vector only (vector); FAK recombinant adenovirus injection (AF), AF plus FAK inhibitor or DMSO. The data are presented as the mean ± SEM (*p < 0.05; **p < 0.01 by two-tailed t test).

To determine whether restoring FAK expression can restore alveolar epithelial integrity after lung damage induced by VILI *in vivo*, we used polyethyleneimine derivative transfection reagent to deliver FAK to the mouse lung via tail vein injection. After FAK delivery, mice underwent HMV for 4 h. Then, 24 h after the experiment, the lung tissues were collected for further investigations. The data indicated that FAK protein levels were significantly increased after injection (Fig. 5A). In addition, we investigated the phosphorylation of Akt using IHC, and the data showed that FAK delivery increased the phosphorylation of Akt in AECs (Fig. 5B). Besides, our study showed that the *in vitro* expression of FAK promoted the phosphorylation of Akt in AECs (Supplemental Fig. 3). Moreover, FAK supplementation significantly improved the integrity of AECs and resulted in lower protein extravasation, lower cell counts in the BALF and a lower lung tissue wet/dry ratio (Fig. 5C–E). HMV significantly increased the level of the alveolar epithelial injury marker RAGE^8^ in the BALF (783.88 pg/ml compared to 285.75 pg/ml in the non-mechanical ventilation control, p = 0.00(F statistic: F = 0.130, p = 0.724)). In contrast, mice in which FAK was pre-delivered exhibited lower RAGE levels in the BALF compared to those in mice in which placebo was delivered, which indicates that FAK can attenuate alveolar injury (Fig. 5F). Furthermore, after HMV, both the pathological injury score and apoptotic index of mouse lung tissues pretreated with FAK were significantly lower than those in the control mice (Fig. 5G,H). However, FAK inhibitor treatment blocked the protective effect of FAK on the pathogenesis of VILI. The mice treated with FAK inhibitor exhibited significantly higher levels of multiple parameters of lung injury, including lung injury score, apoptosis index, BALF protein level, BALF cell counts, and the RAGE level in the BALF, than those exhibited by the mice treated with FAK alone, (Fig. 5C–H).

## Discussion

In this study, we utilized both *in vitro* and *in vivo* approaches to investigate the effect of VILI on AECs. We present compelling results that VILI damages AECs and induces the loss of alveolar integrity. Our study found that VILI decreases FAK expression, while increasing FAK expression can attenuate VILI by facilitating the repair of epithelial cells. In addition, the protective effect of FAK may occur via the FAK-Akt pathway.

FAK is known as a nonreceptor tyrosine kinase that can be activated by autophosphorylation with conformational changes, and then phosphorylate other kinases, and plays roles in various cell events. It is known that mechanical stress, such as mechanical stress in cardiac myocytes^9,10^, shear stress in fibroblasts^11^, and stretch in endothelial cells^12^ can modulate FAK expression in cells. Liu XY *et al*. found that FAK is crucial for type 2 AEC survival during tonic stretch^13^, while expression of FAK promotes the migration of type 2 AECs^14^. In another study, the FAK phosphorylation level was decreased when type 2 AECs were mechanically dissociated from the matrix *in vitro*^15^. Moreover, FAK plays an important role in the pathogenesis of lung injury. Desai LP *et al*. suggested that HMV induces lung injury and decreases the level of FAK phosphorylation, which could determine the fate of AECs^6^. In a study on pulmonary fibrosis, Qiang D *et al*. found that blocking FAK can trigger the apoptosis of type 2 AECs^5^. Furthermore, FAK knockout makes mice susceptible to lung injury. In a study by Wheaton AK *et al*., the deletion of FAK increased sensitivity to apoptosis in lung epithelial cells, and the anti-apoptotic effect of FAK occurred via blocking caspase-8^7^. Therefore, FAK may be involved in VILI by contributing to epithelial cell survival. In our study, we demonstrated decreased FAK expression in AECs that underwent VILI. FAK is shown to be upstream of the PI3K-Akt pathway, and FAK augmented the phosphorylation of the p85 subunit of PI3K and Akt and then protected cells from apoptosis^16^. Therefore, decreased FAK expression decreases the activity of the PI3K-Akt pathway. Blocking PI3K-Akt inhibits cell proliferation and migration and sensitivity to apoptosis. During the pathogenesis of VILI, the mechanical stretching of AECs decreased the expression of FAK and downstream PI3K-Akt, which induced apoptosis of AECs, inhibits the proliferation of cells, and then destroy the integrity of alveoli. In contrast, the enhanced expression of FAK in AECs may maintain the activation of the PI3K-Akt pathway and lung function.

However, although our study showed the role of FAK in VILI, the potential pathway that regulates FAK in alveolar epithelial cells remains unknown. SRY-related HMG-box (SOX) proteins are known as transcription factors that contain subtypes of high-mobility-group domains and can bind to specific sequences in the minor groove of DNA. SOX11 knockout mice display hypoplasia of the lung^17^. In early 2019, we reported that the expression of both Sox11 and FAK in alveolar epithelial and interstitial cells is significantly downregulated in VILI and that the expression of these molecules is correlated^18^. In addition, the effect of FAK on the physiology of the lung remains controversial. For example, in another study, Lederer PA *et al*. suggested that a FAK antagonist improves the recovery of vascular endothelial cells during lung injury induced by endotoxins^19^. However, this study did not evaluate pathological changes in AECs in lung injury. We speculate that, compared to VILI, endotoxins can induce a more intensive inflammatory response and then destroy alveolar and vascular cells, while a FAK antagonist can block multiple downstream effects of FAK, including PI3K-Akt^20^ and then inhibit the inflammatory response, which facilitates recovery. In our study, we found that FAK expression contributed to the repair of AECs, which indicates multiple roles of FAK in the pathogenesis of lung injury. However, besides AECs, some other types of cells in the lungs may express FAK under physiological or pathological condition. Researchers have also found that mechanical stress can facilitate lung fibrosis via activated FAK^21^, which is consistent with the complicity of FAK. FAK expression and activity are upregulated by TGF-β1in fibroblast foci and remodelled vessels from lung fibrosis patients^22^, and then induce myofibroblast differentiation and collagen deposition. Conversely, Ghosh MC *et al*. argue that migrating cells have decreased levels of FAK phosphorylation^14,23^. Therefore, we speculate that FAK may have different effects in different types of cells^7^. More research is needed to understand the role of FAK in lung injury. And Yalcin HC *et al*. designed a cell culture model for simulating cyclic airway closure/reopening *in vitro*, which can be used in studies to directly investigate role of FAK expression during the pathogenesis of lung injury^24^.

In conclusion, we have identified a novel mechanism by which high volume mechanical ventilation injures alveolar epithelial cells and then downregulates FAK levels. The decreased FAK level impairs the regeneration of alveolar epithelial cells. Moreover, the delivery of FAK to AECs can reduce lung injury in response to VILI.

## Material and Methods

### Experimental VILI

Male C57BL/6 mice aged 10 weeks were purchased from The Experimental Animal Center of Beijing University of Medical Sciences (Beijing, China), and all animal care procedures and experiments were conducted according to the ARRIVE guidelines and approved by the Committee of Ethics on Animal Experiments of Hebei Medical University. The mice were anaesthetized with an intraperitoneal injection of 3% pentobarbital sodium (50 mg/kg body weight). During mechanical ventilation, additional doses of intraperitoneal pentobarbital sodium were given depending on the depth of anaesthesia and the duration of the experiments. The mice were intubated with a 21 G steel endotracheal cannula via tracheostomy and connected to a rodent ventilator (Shenzhen, China). Then, mechanical ventilation was applied with room air, with a tidal volume of 30 ml/kg, a respiratory rate of 75 breaths/min, and a positive end-expiratory pressure of 0 cm H2O for 4 h^25^. The mice were randomly divided into the following groups (8 mice per group): the non-mechanical ventilation control group; the HMV group; the HMV + FAK delivery group; the HMV + vehicle delivery group; the HMV + FAK delivery + FAK inhibitor (PF562271, purchased from Selleckchem, 25 mg/kg, dissolved in DMSO) group^26^; and the HMV + FAK delivery + DMSO group. In the HMV group, mice underwent mechanical ventilation; in the HMV + FAK delivery/vehicle delivery/FAK inhibitor/DMSO group, the mice received FAK recombinant adenovirus, vehicle, FAK inhibitor, or DMSO injection via the tail vein before mechanical ventilation. In the control group, the mice underwent anaesthesia and endotracheal cannula via tracheostomy but did not undergo mechanical ventilation. Twenty-four hours after the experiment, the mice were euthanized, and the bronchoalveolar lavage fluid (BALF) from the left lung and lung tissues from the right side were collected^27^.

### Cell culture

Both MLE-15 and 293 T cells were purchased from the American Type Culture Collection and were maintained in HITES medium or DMEM (Sigma) supplemented with ultracentrifuged FBS (Invitrogen) at 37 °C in a humified atmosphere of 5% CO2. MLE-15 cells were treated with a FAK inhibitor (PF562271, dissolved in DMSO) at a concentration of 1.5 nM^28^.

### Plasmid construction and transfection

Mouse FAK cDNA (Origene) were sequenced and subcloned into the pCMV-IRES-EGFP plasmid (Addgene), and the IRES-EGFP sequence was removed to create pCMV-FAK following the manufacturer’s instructions. Then, 293 T cells were transfected with adenoviral vector and pCMV-FAK using the calcium phosphate precipitation method, and FAK recombinant adenovirus (AF) was harvested from the cell lysate. MLE-15 cells were transfected using transfection reagent. Twenty-four hours after transfection, western blotting was performed to validate the transfection, and then the cells were used for other experiments.

To overexpress FAK *in vivo*, FAK recombinant adenovirus (5.0 × 10^10^ pfu) was diluted in sterile saline and then injected into the tail vein of mice. The transfection efficiency in the lungs was evaluated by immunochemistry.

### Bronchoalveolar lavage fluid collection and analysis

The left lung was slowly infused with 1.5 ml of PBS (4 °C) and washed three times (0.5 ml of PBS, 1 min each time) to obtain the bronchoalveolar lavage fluid. Then, the fluid was centrifuged at 1500 × g for 10 min at 4 °C to collect cells, and the supernatant was centrifuged again at 12000 × g for 10 min at 4 °C to collect protein. In addition, the protein concentration was measured using a detergent-compatible BCA protein assay (Bio-Rad) according to the manufacturer’s instructions. The cell pellets collected from the BALF samples were resuspended in 1 ml of PBS. The total number of nucleated cells in the BALF was counted with a haemocytometer.

### Lung wet-to-dry (W/D) weight ratio

The gross vessels of the right lung were removed. The middle lobe of the right lung was excised, and the wet weight was measured. Subsequently, the middle lobe of the right lung was placed in an oven at 60 °C for 3 days and then weighed to determine the dry weight. After all the measurements, the W/D weight ratio was calculated. In addition, the lower lobe of the right lung was infiltrated with 2% paraformaldehyde for histological analysis. The upper lobe of the right lung was frozen at −80 °C for further biochemical assays.

### Histological evaluation and immunochemistry (IHC)

The lower lobe of right lung tissue was fixed, embedded in paraffin, and then cut into sections (5 μm). For histological evaluation, the slides were stained with haematoxylin and eosin and examined separately by two pathologists who were blinded to the study conditions. Lung injury was scored based on the following features, as described in a previous study^29^: (1) haemorrhage, (2) alveolar congestion, (3) the thickness of the alveolar wall, and (4) the infiltration of neutrophils into the airspace or the vessel wall. Each feature was scored based on the following scale: 0, minimal (little) damage; 1, mild damage; 2, moderate damage; 3, severe damage; and 4, maximal damage.

For IHC, lung tissue sections on glass slides were deparaffinized, rehydrated and then subjected to antigen retrieval. Thereafter, the slides were blocked with goat serum and incubated with rabbit monoclonal antibodies (1:200 dilution) against FAK and phospho-Akt (Abcam) and then with a horseradish peroxidase-labelled anti-rabbit antibody (Abcam). Finally, the slides were developed with diaminobenzidine and counterstained with haematoxylin. Target protein expression was scored semiquantitatively according to the regular IHC staining grade system, and the values were used for statistical analysis.

### Cell stretch (CS) experiments *in vitro*

CS experiments were performed using a Flexercell FX-4000T cell stretch system equipped with a 25-mm BioFlex loading station (Flexcell International). The cells (10^6^) were mounted in a Flexercell FX-4000T Strain Unit and then exposed to 18% CS for 4 h (30 cycles per min) to recapitulate high-tidal volume mechanical ventilation regimen-induced cell injury^30^. Control cells were also placed in the Flexercell Strain Unit and underwent all processes except stretch treatment. At the end of the experiment, the cells were collected for future analysis.

### Western blotting

Lung tissues or cell pellets were lysed in RIPA buffer containing proteinase inhibitor. Equal amounts of protein (20 μg) were loaded on 8–10% SDS-PAGE gels and then electro-transferred onto PVDF membranes (Millipore Corp). The membranes were then blocked with BSA and incubated with the indicated primary antibody (1:3,000) overnight at 4 °C. Subsequently, the membranes were incubated with a secondary antibody (1:3,000, Abcam, anti-rabbit IgG). Then, the protein level on the blot was detected using the Western Bright ECL kit (Bio-Rad Laboratories). Equal loading of the samples was validated by the detection of GAPDH. The following antibodies against the target proteins in the study were used: rabbit monoclonal anti-FAK (Abcam), rabbit polyclonal anti-phospho-FAK (at Tyr397, Cell Signalling), mouse monoclonal anti-phosphated-Akt (at Ser 473, Santa cruz), and rabbit monoclonal anti-GAPDH (Abcam).

### siRNA transfection

Cells were seeded in 12-well culture plates (10^5^ cells/well) and then transfected with 40 nM anti-FAK siRNA or a scrambled probe (Santa Cruz) using Lipofectamine 2000 (Invitrogen) according to the manufacturer’s instructions. Twenty-four hours after transfection, western blotting was performed to validate the results of inhibition, and then the cells were used for other experiments.

### Apoptosis assay

A TUNEL assay was applied to evaluate the apoptosis of AECs in the lung. In brief, paraffin sections were dewaxed, and antigen retrieval was performed. Then, the slides were incubated a mixture of TdT and biotin-dUTP reagent according to the manufacturer’s protocol (TUNEL kit, Sigma). Then, the tissues were stained with streptavidin-HRP and DAB substrate. The apoptosis of epithelial cells was analysed by light microscopy.

Flow cytometry was applied to evaluate the apoptosis of cells *in vitro*. After CS treatment, cells were collected and washed with PBS and then resuspended in 500 μl of binding buffer containing 5 μl annexin V-FITC and 10 μl of propidium iodide (PI) (Bio-Rad). After incubation for 30 min in the dark at room temperature, the apoptosis ratio was measured by a FACScan flow cytometer (Becton Dickinson) according to the instructions provided with the annexin V/PI kit.

### ELISA

The concentration of receptor for advanced glycation end product (RAGE) in the BALF was quantified by ELISA according to the manufacturer’s instructions (Abcam). Briefly, biotinylated RAGE antibody was added to the plate, and then BALF was added to the wells. Standard RAGE reagents were diluted and added to the wells to generate a standard curve. After 2 h of incubation at room temperature, the absorbance was read at 450 nm using a Multimode Plate Reader.

### Cell viability assay

In the cell viability assay, cells were seeded in 96-well plates (5000 cells/well). Then, the cells were cultured for 72 h. Subsequently, cell viability was measured via a WST-1 assay (Roche Diagnostics) according to the protocol. The absorbance was read at 450 nm using a Multimode Plate Reader.

### Wound healing assay

Cells were seeded in 12-well plates after various treatments and then cultured until confluence. Next, wounds were inflicted using a sterile pipette tip to scratch a straight line, and the plates were washed with PBS to remove the debris. The remaining cells were allowed to grow continuously in the wells. The gap distance was quantitatively evaluated and photographed at 0 and 72 h.

### Primary AEC collection

Mice was euthanized, and then the lung tissues were collected, perfused and lavaged. The gross vessels of the lung were removed. Then, the lung tissues were instilled with 2 ml of dispase followed by 0.5 ml of liquefied agarose and incubated at room temperature for 1 h. Subsequently, the tissue and cell mixture were washed, collected and filtered with 100-μm pore, 70-μm, 48-μm and 30-μm nylon mesh. The filtrate was immersed in DMEM at room temperature and then centrifuged for 15 min at 160 × g at 4 °C to collect the cell pellet. Finally, the cells were stained with anti-mouse streptavidin-PE (Biolegend) and EpCAM-APC (Thermo Fisher) at 4 °C for 1 h and resuspended in buffer with DAPI. The mixed cells were sorted using a FACS Aria cell sorter (Becton Dickinson), and primary murine AECs were collected.

### Statistics

All *in vitro* data are presented as experiments of triplicates with the mean ± SEM. Two-tailed t test and ANOVA used for the statistical analysis. P values less than 0.05 were considered significant.

## Supplementary information

Supplementary Information 
