# Supplementary material for: Enhancement of FAK alleviates ventilator-induced alveolar epithelial cell injury
Source: Sci Rep. 2020 Jan 15;10:419. doi: 10.1038/s41598-019-57350-6 (PMC6962166; doi:10.1038/s41598-019-57350-6)

**Enhancement of FAK alleviates ventilator-induced alveolar epithelial cell injury**

Mingxing Fang, Na Liu, Xiaoguang Yao, Tieling Xu, Zhiyong Wang

**Supplement figures**

**
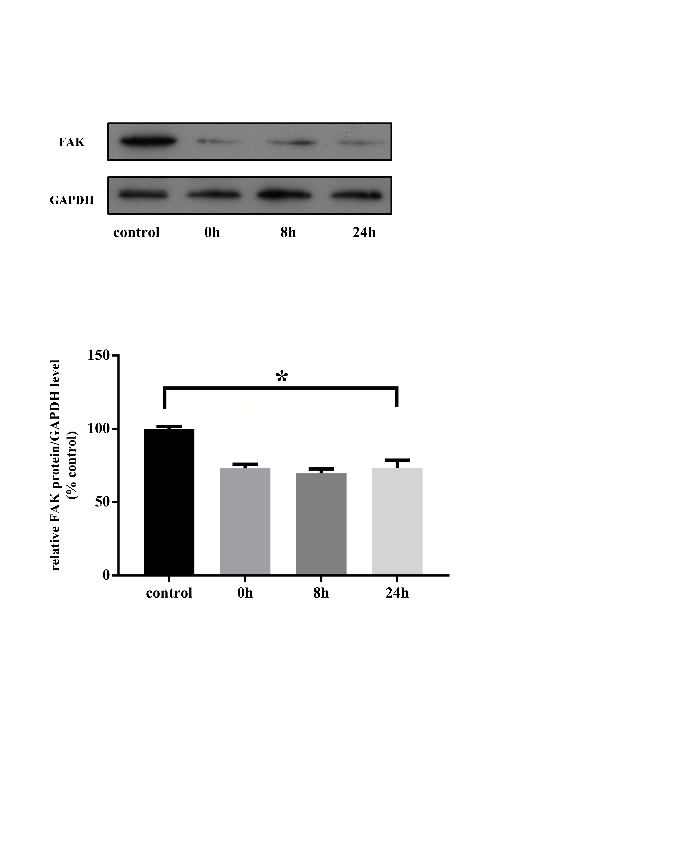
**

**Supplemental Figure 1** FAK expression in AECs at different timepoints after HMV treatment in mice. Lung tissues were collected 0, 8, and 24 h after HMV treatment, and then AECs were sorted using flow cytometry. Thereafter, protein was collected, and FAK expression was evaluated by western blotting. The data showed that FAK expression was decreased significantly in the treatment group compared to the untreated control group, whereas there was no significant difference in FAK expression among the different timepoints (representative images and relative quantifications are shown). All experiments were performed in triplicate, and the data are presented as the mean ± SEM (*, p<0.05; **, p<0.01 by ANOVA).


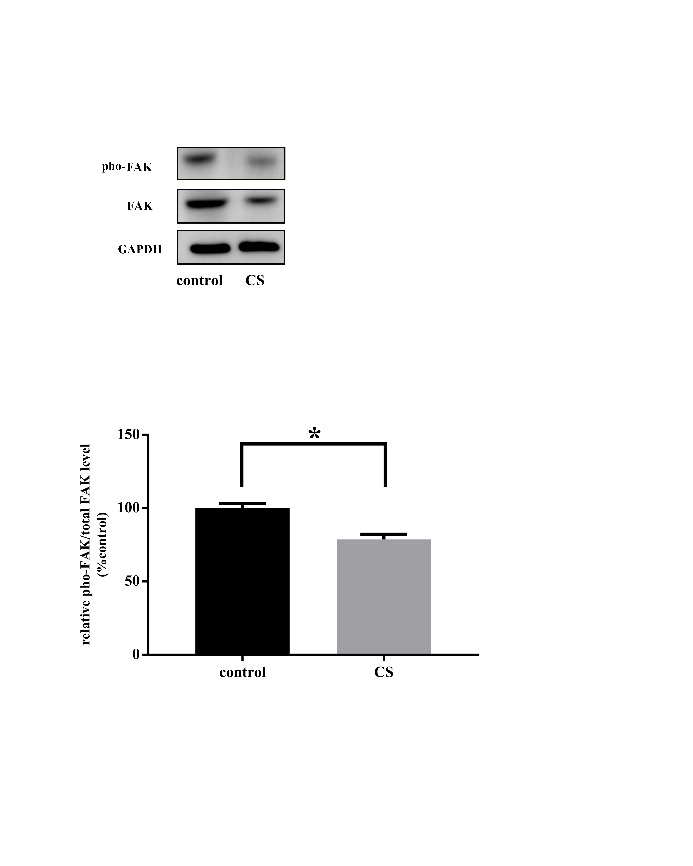


**Supplemental Figure 2** Decreased phosphorylated FAK expression in AECs under CS. AECs were received CS treatment as described in the methods, and phospho-FAK (at Tyr397) expression was evaluated by western blotting. The data showed that the expression of phospho-FAK was decreased significantly compared to that in the controls (representative images and relative quantifications are shown). All experiments were performed in triplicate, and the data are presented as the mean ± SEM (*, p<0.05; **, p<0.01 by t test by two-tailed t test).


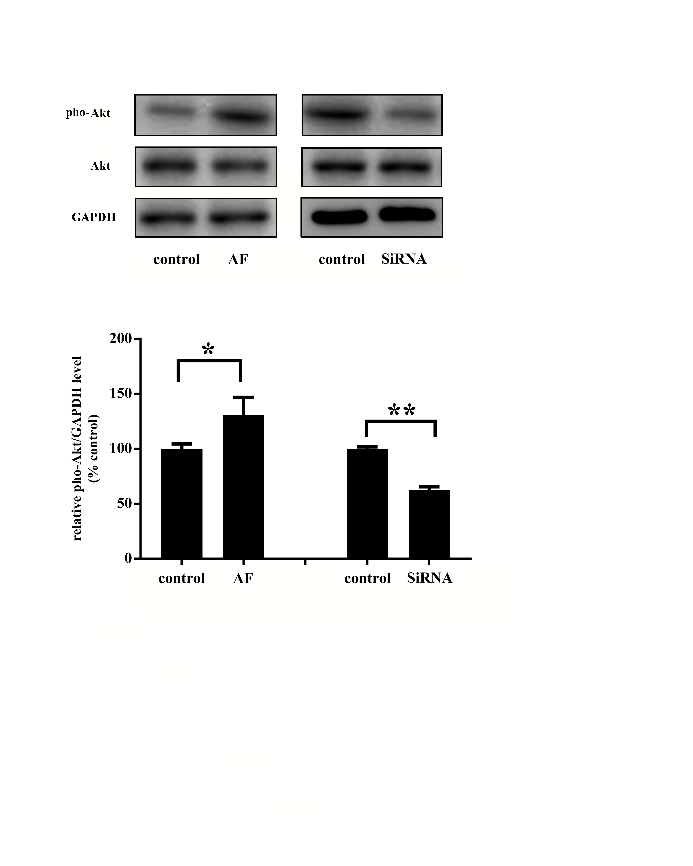


**Supplemental Figure 3** FAK expression increases the level of Akt phosphorylation in AECs. In AECs with FAK expression enhancement or knockdown, phospho-Akt expression was evaluated by western blotting, and the results showed that the level of phosphorylation of Akt was significantly increased in cells transfected with AF and was decreased in FAK knockdown cells (representative images and relative quantifications are shown). All experiments were performed in triplicate, and the data are presented as the mean ± SEM (*, p<0.05; **, p<0.01 by two-tailed t test).

**Complete original Western blots showing staining with antibodies against GAPDH or FAK**




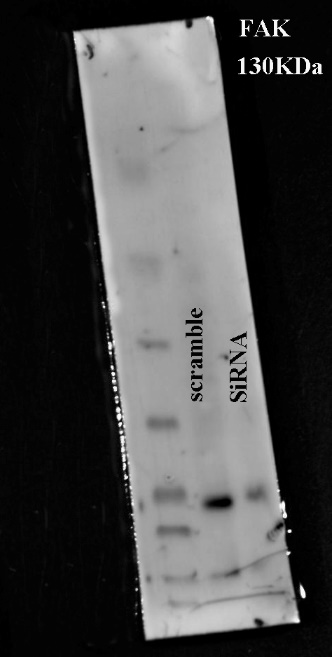

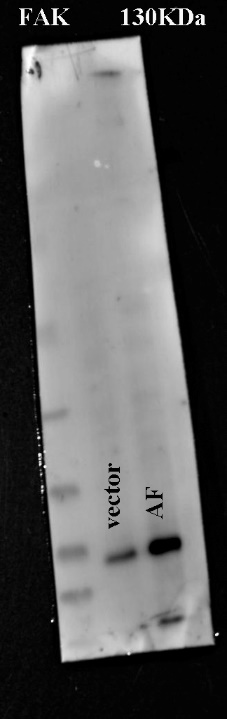

Supplement: Supplementary file 1 — Supplementary Information [file 41598_2019_57350_MOESM1_ESM.docx]
